# ANKFY1 bridges ATG2A-mediated lipid transfer from endosomes to phagophores

**DOI:** 10.1038/s41421-024-00659-y

**Published:** 2024-04-16

**Authors:** Bin Wei, Yuhui Fu, Xiuzhi Li, Fang Chen, Yiqing Zhang, Hanmo Chen, Mindan Tong, Linsen Li, Yi Pan, Shen Zhang, She Chen, Xiaoxia Liu, Qing Zhong

**Affiliations:** 1 Key Laboratory of Cell Differentiation and Apoptosis of Chinese Ministry of Education, Department of Pathophysiology, Shanghai Jiao Tong University School of Medicine, Shanghai, China; 2 National Institute of Biological Sciences, Beijing, China

**Keywords:** Macroautophagy, Membrane trafficking, Autophagosomes

## Abstract

Macroautophagy is a process that cells engulf cytosolic materials by autophagosomes and deliver them to lysosomes for degradation. The biogenesis of autophagosomes requires ATG2 as a lipid transfer protein to transport lipids from existing membranes to phagophores. It is generally believed that endoplasmic reticulum is the main source for lipid supply of the forming autophagosomes; whether ATG2 can transfer lipids from other organelles to phagophores remains elusive. In this study, we identified a new ATG2A-binding protein, ANKFY1. Depletion of this endosome-localized protein led to the impaired autophagosome growth and the reduced autophagy flux, which largely phenocopied ATG2A/B depletion. A pool of ANKFY1 co-localized with ATG2A between endosomes and phagophores and depletion of UVRAG, ANKFY1 or ATG2A/B led to reduction of PI3P distribution on phagophores. Purified recombinant ANKFY1 bound to PI3P on membrane through its FYVE domain and enhanced ATG2A-mediated lipid transfer between PI3P-containing liposomes. Therefore, we propose that ANKFY1 recruits ATG2A to PI3P-enriched endosomes and promotes ATG2A-mediated lipid transfer from endosomes to phagophores. This finding implicates a new lipid source for ATG2A-mediated phagophore expansion, where endosomes donate PI3P and other lipids to phagophores via lipid transfer.

## Introduction

Macroautophagy (hereafter autophagy) is a degradative pathway characterized by de novo formation of double-membraned, cellular material-containing vesicles termed autophagosomes^1^. During autophagosome biogenesis, a flattened membrane structure named phagophore is first formed, which then expands and bends to engulf a portion of cytoplasm, before enclosure to give rise to an autophagosome. The autophagosome would eventually fuse with lysosome, where its cargoes and inner membranes are degraded^2^. The process of autophagosome biogenesis is driven by a series of ATG (autophagy-related gene) proteins, also termed core autophagy machinery, that forms several functional complexes and functions in a hierarchal way, as reviewed elsewhere^2–6^. Briefly, the ULK1 complex phosphorylates and activates the PI3KC3C1 complex, which generates PI3P on initial membrane. Then, PI3P recruits WIPIs, which in turn recruit more autophagy machinery proteins for autophagosome formation.

It is estimated that autophagy probably needs to mobilize millions of lipid molecules per cell for phagophore membrane extension while engulfing substrates upon autophagy stimulation^7^. So far, three distinct mechanisms for delivery of lipids for phagophore expansion have been proposed: vesicle-mediated delivery, membrane extrusion from pre-existing organelles, and protein-mediated lipid transport^7^. For protein-mediated lipid transport, lipids can be transferred directly by lipid transfer protein at one or more putative contact sites in close proximity between phagophores and other organelles (e.g., endoplasmic reticulum (ER)). There are four lipid transfer proteins implicated in autophagosome biogenesis^5^, including ATG2, GRAMD1A (GRAM domain-containing 1 A), VPS13A, and TipC (putative vacuolar protein sorting-associated protein 13 C), among which, ATG2 is the most studied one.

*ATG2* was first identified alongside 12 other *ATG* genes from a screen of autophagy-defective mutants in *Saccharomyces cerevisiae*^8^. Its function in autophagosome formation was demonstrated in both yeast^9^ and mammal^10^. There are two isoforms of mammalian ATG2, ATG2A and ATG2B, which are functionally redundant to promote autophagic degradation^10^. Both isoforms have an N-terminal Chorein domain with a longitudinal hydrophobic groove that binds to lipids with little or no lipid specificity^11^. The membrane curvature determines membrane binding ability of ATG2^12^. It was reported that ATG2A binds tightly to and tethers small unilamellar vesicles (~30 nm), but interacts weakly with and does not tether large unilamellar vesicles (LUVs, ~100 nm)^12,13^. WIPI4 can form a complex with ATG2A, and enhance the membrane tethering ability of ATG2 for PI3P-positive LUVs^12,13^. Both mammalian ATG2A and ATG2B can transfer lipids between tethered membranes in vitro^11,13^. It is widely accepted that ATG2 facilitates lipid transfer between ER and growing phagophores during the early stages of autophagosome formation^14,15^, where the membrane contact with phagophore is stabilized by interaction with PI3P effector protein WIPI4 on the phagophore^12^, and the membrane anchoring to ER is accomplished by interactions with ER-resident scramblases^16^. Many different organelles have been implicated as potential membrane sources supporting autophagosome growth^17^. However, so far it is not clear whether ATG2 can transfer lipids from other organelles to phagophores.

In this study, we identified Ankyrin Repeat and FYVE Domain Containing 1 (ANKFY1) as an interaction partner of mammalian ATG2A. ANKFY1 is an endosomal membrane protein with limited known functions in autophagosome biogenesis. Depletion of ANKFY1 resulted in the impaired phagophore expansion and the reduced autophagy flux, which phenocopied the silencing of ATG2A/B. A pool of ANKFY1 co-localized with ATG2A between endosomes and phagophores upon Torin 1 treatment. In vitro experiments using liposomes suggested that ANKFY1 facilitates the lipid transfer by ATG2A through enhancing the membrane tethering ability of ATG2A for PI3P-positive liposomes. Depletion of either ATG2A/B, ANKFY1, or UVRAG reduced the amount of PI3P on phagophores, suggesting that endosomes provide PI3P for phagophore expansion. In summary, we propose that ATG2A transfers lipids including PI3P from endosomes to phagophores, with ANKFY1 bridging its interaction with endosomes.

## Results

### ANKFY1 interacts with ATG2A upon autophagy stimulation

**Fig. 1:**
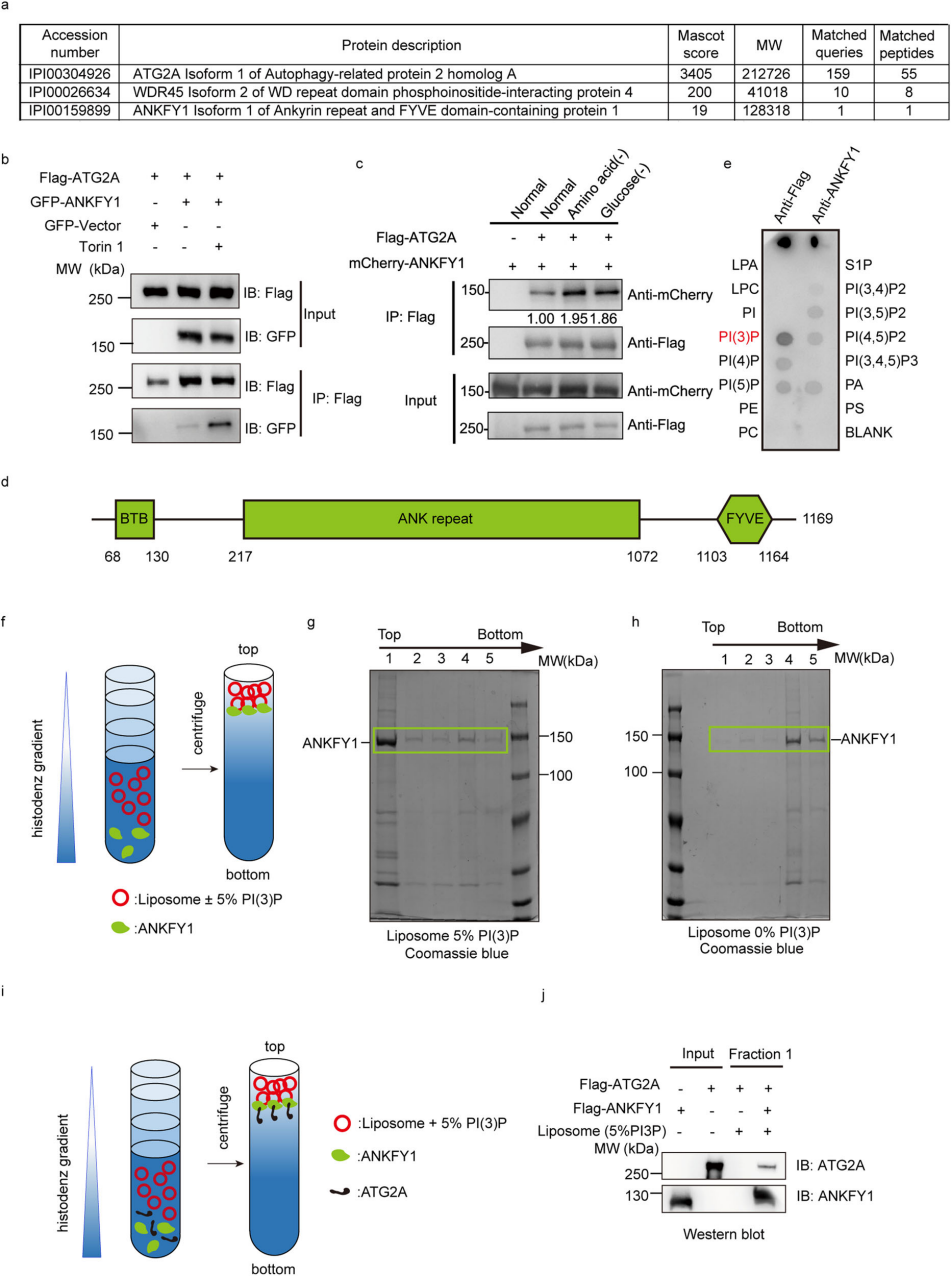
ANKFY1 interacts with ATG2A. **a** Tandem affinity purification of ATG2A identify ANKFY1 as a ATG2A interaction protein. Proteins shown in the table were identified by MS from purified complexes. **b** Verification of protein interaction between ATG2A and ANKFY1. GFP-labeled ANKFY1 and Flag-labeled ATG2A were co-expressed in HEK293T cells with or without 200 nM Torin 1 treatment for 3 h. Cell lysates were immunoprecipitated (IP) with anti-Flag antibody. **c** HEK293T cells transiently expressing mCherry-ANKFY1 and Flag-ATG2A were exposed to regular culture conditions, amino acid starvation, or glucose starvation for 6 h. Subsequently, cell lysates were subjected to immunoprecipitation using an anti-Flag antibody. **d** Schematic representation of the domain organization in ANKFY1. **e** Purified Flag-ANKFY1 protein was incubated with the lipid strip containing biologically important lipids and probed with anti-Flag antibody. Flag-ANKFY1 protein was blotted on strip and stained with anti-Flag and anti-ANKFY1 antibodies as a positive control. **f** Schematic diagram outlining liposome co-floatation assay. Liposomes were incubated with ANKFY1 and then purified by floatation on a three-layer histodenz gradient (35%, 25%, and 0%). **g**, **h** Liposome flotation assay fractions were evaluated by SDS-PAGE and Coomassie blue staining for reactions using liposomes containing 2% Rhod-PE, 30% POPE, 63% DOPC and 5% PI3P (**g**) or 2% Rhod-PE, 30% POPE, 68% DOPC and 0% PI3P liposomes (**h**). **i** Schematic diagram outlining liposome co-floatation assay. Liposomes containing 2% Rhod-PE, 30% POPE, 63% DOPC and 5% PI3P were incubated with ATG2A and ANKFY1 as indicated. The incubation mixture was purified by floatation on a three-layer histodenz gradient (35%, 25%, and 0%) and the topmost fraction was collected and analyzed by SDS-PAGE. **j** The topmost fraction in **i** was analyzed by SDS-PAGE and western blot.

To dissect how ATG2A transfers lipids for phagophore expansion, we performed tandem affinity purification of ZZ-Flag-ATG2A in U_2_OS cells to find its binding proteins. Among the eluted proteins identified by mass spectrometry (MS), we found WIPI4, a previously reported interaction protein of ATG2A. Besides, an endosome-localized protein ANKFY1^18^ caught our attention (Fig. 1a). We performed co-immunoprecipitation experiments in HEK293T cells to verify the interaction between ATG2A and ANKFY1. GFP-ANKFY1 co-precipitated with Flag-ATG2A and their interaction was enhanced by addition of Torin 1, a widely used autophagy stimulator (Fig. 1b). This enhancement of interaction was also observed under conditions of glucose or amino acid starvation (Fig. 1c). As both of these two proteins were reported to localize on membrane, with ATG2 localizing on phagophores and ANKFY1 on endosomes, we wondered whether ANKFY1 can bind to ATG2A on the membrane. Domain analysis indicated that ANKFY1 contains a FYVE domain (Fig. 1d)^18^, which is a well-known PI3P-binding domain. We performed the protein–lipid overlay assay by commercial lipid strip to examine whether purified ANKFY1 can bind PI3P. The result showed that ANKFY1 binds PI3P and this binding is much stronger than binding to other PIs (Fig. 1e; Supplementary Fig. S1a). By using co-floatation assay (Fig. 1f), we validated the binding ability of ANKFY1 to PI3P-containing liposomes (100 nm in size) (Fig. 1g, h). Besides, co-floatation assay showed weak binding of ATG2A to PI3P-containing liposomes with 100 nm in size, where ATG2A was not enriched on the topmost layer (strong binding to vesicles), but in the middle of the histodenz gradient (weak to median binding to vesicles) (Supplementary Fig. S1b–d). Next, we examined whether ATG2A can be harvested on the topmost layer with PI3P liposomes with ANKFY1 (Fig. 1i). The results showed that ATG2A alone was not detected on the topmost fraction (Fig. 1j), but ATG2A was able to be recruited to the PI3P liposomes on the topmost fraction in the presence of ANKFY1 (Fig. 1j). We performed the assay reciprocally, where ATG2A was tethered to DGS-NTA-containing liposomes through the specific interaction between the His tag and DGS-NTA lipid. Similarly, anchoring ATG2A-His to liposomes containing 5% DGS-NTA resulted in the enrichment of ANKFY1 on the liposomes (Supplementary Fig. S1e, f). These findings imply that the membrane facilitates the interaction between ATG2A and ANKFY1, pointing a high likelihood of their interaction on membranes.

### Depletion of ANKFY1 reduces the autophagy flux

**Fig. 2:**
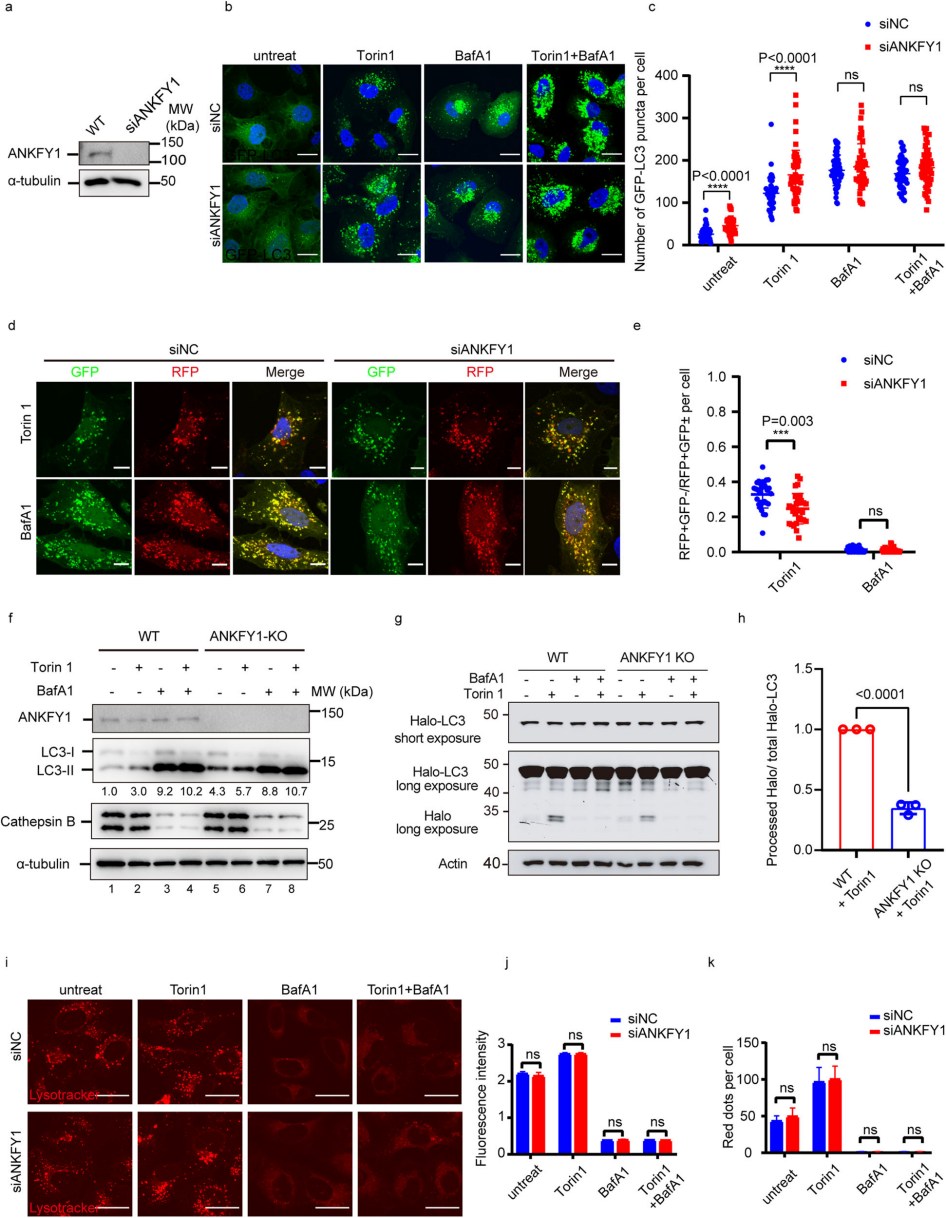
Depletion of ANKFY1 reduces the autophagy flux. **a** The knockdown efficiency of ANKFY1 in U_2_OS cells. Cell lysates were analyzed by immunoblotting with indicated antibodies. **b** U_2_OS cells stably expressing GFP-LC3 were transfected with non-target control siRNA or ANKFY1 siRNA and then cultured in regular DMEM. 48 h after transfection, cells were treated with 200 nM Torin 1, 100 nM BafA1, or both for 3 h, fixed, stained with DAPI and imaged under confocal microscope. Scale bars, 10 μm. **c** Quantification of the results in **b**. Data are mean ± SD for 50 cells. ****P ≤ 0.0001; ns, not significant. **d** U_2_OS cells stably expressing GFP-mRFP-LC3 were transfected with non-target control siRNA or ANKFY1 siRNA and then cultured in regular DMEM. 48 h after transfection, cells were treated with 200 nM Torin 1, 100 nM BafA1, or both for 3 h, fixed, stained with DAPI and imaged under confocal microscope. Scale bars, 10 μm. **e** Quantification of the results in **d**. Data are mean ± SD for 60 cells. ***P ≤ 0.001; ns, not significant. **f** Autophagic flux is decreased in ANKFY1 KO cells. WT and ANKFY1 KO U_2_OS cells were treated with 200 nM Torin 1, 100 nM BafA1, or both for 3 h, and then immunoblotting was performed with the indicated antibodies. **g** In-gel fluorescence detection of total cell lysates from WT and ANKFY1 KO U_2_OS cells infected with lentivirus expressing Halo-LC3B, pulse-labeled with 100 nM TMR-conjugated Halo ligand for 30 min, and then treated with Torin 1 (200 nM, 3 h), BafA1 (100 nM, 3 h), or both. **h** Quantification of results shown in **g**. Processed Halo band intensity (long exposure) was first divided by corresponding band intensity of Halo-LC3B (short exposure) to obtain processed Halo/total Halo-LC3 ratio, which are then normalized to sample (WT + Torin1). Data are mean ± SD from three independent experiments. Statistical significance was determined by two-tailed *t*-test. *P-*value is listed. **i** U_2_OS cells were transfected with non-target control siRNA or ANKFY1 siRNA and then cultured in regular DMEM. 48 h after transfection, cells were treated with 200 nM Torin 1, 100 nM BafA1, or both for 3 h. Cells were stained by 5 nM LysoTracker for 30 min, and imaged under confocal microscope. Scale bars, 30 μm. **j**, **k** Quantification of the results in **i**. Data are mean ± SD for 60 cells. ns, not significant.

It is well-known that ATG2 is a key regulator of phagophore expansion. We wondered the role of ANKFY1 as a binding partner of ATG2 in autophagy. To address this, we first quantified microtubule-associated protein light chain 3 (LC3)-II turnover by fluorescence microscopy in U_2_OS cells stably expressing GFP-LC3 upon ANKFY1 depletion. The knockdown efficiency of ANKFY1 by siANKFY1 was confirmed by western blot (Fig. 2a). Upon treatment with V-ATPase inhibitor Bafilomycin A1 (BafA1), GFP-LC3 puncta were accumulated to the same level in siNC and siANKFY1 U_2_OS cells (Fig. 2b, c). However, compared to siNC cells, knockdown of ANKFY1 increased the number of GFP-LC3 puncta in both untreated condition and Torin 1-treated condition (Fig. 2b, c). This accumulation is likely due to the impaired delivery or degradation of LC3-II in lysosomes upon ANKFY1 depletion. Moreover, we analyzed the autophagy flux using the reporter GFP-mRFP-LC3, where LC3 is tagged with acid-resistant monomeric red fluorescent protein (mRFP) and acid-sensitive green fluorescent protein (GFP). The results showed that the ratio of RFP^+^GFP^–^ puncta to total RFP^+^ puncta was reduced upon ANKFY1 depletion in Torin 1-treated condition (Fig. 2d, e), which suggests that autophagy flux is reduced in ANKFY1-depleted cells.

To further support our hypothesis, we generated ANKFY1 knockout (KO) U_2_OS cells by CRISPR/Cas9-mediated genome editing method. ANKFY1 was successfully knocked out as evidenced by western blot (Fig. 2f). We then compared the autophagy flux between ANKFY1 wild-type (WT) and KO U_2_OS cells by examining LC3-II turnover by western blot. LC3-II accumulated to the same level upon BafA1 treatment in ANKFY1 KO and WT cells (Fig. 2f, lane 7 vs lane 3, and lane 8 vs lane 4), suggesting that ANKFY1 depletion did not affect the lipid conjugation of LC3. Additionlly, knockout of ANKFY1 caused the accumulation of LC3-II in both untreated condition (Fig. 2f, lane 5 vs lane 1) and Torin 1-treated condition (Fig. 2f, lane 6 vs lane 2). Furthermore, we performed the Halo-LC3 turnover assay^19^ to compare autophagy flux between ANKFY1 WT and KO cells. Briefly, Halo tag becomes resistant to proteolysis after covalently binding to its ligand TMR and persists in lysosomes. Therefore, autophagic flux can be quantitatively determined by in gel fluorescence intensity of TMR-Halo. As expected, the depletion of ANKFY1 decreased the Halo-LC3 degradation, as evidenced by weaker fluorescence intensity of TMR-Halo band on SDS-PAGE (Fig. 2g, h).

Lastly, we examined whether lysosomal acidity and function is affected in ANKFY1-depleted cells. The cells were stained with acidic organelle-specific fluorescence dye LysoTracker for lysosomal acidity analysis (Fig. 2i). The results showed that neither LysoTracker intensity (Fig. 2j) nor the the number of puncta (Fig. 2k) changed significantly upon ANKFY1 depletion. We examined the maturation of cathepsin B by western blot. The matured form of cathepsin B was reduced by BafA1 treatment but not by the depletion of ANKFY1 (Fig. 2f). These data suggest that ANKFY1 is not likely to be essential for the lysosomal activity. Taken together, ANKFY1 depletion reduces the autophagy flux but not lysosome functions.

### ANKFY1 is important for autophagosome expansion

**Fig. 3:**
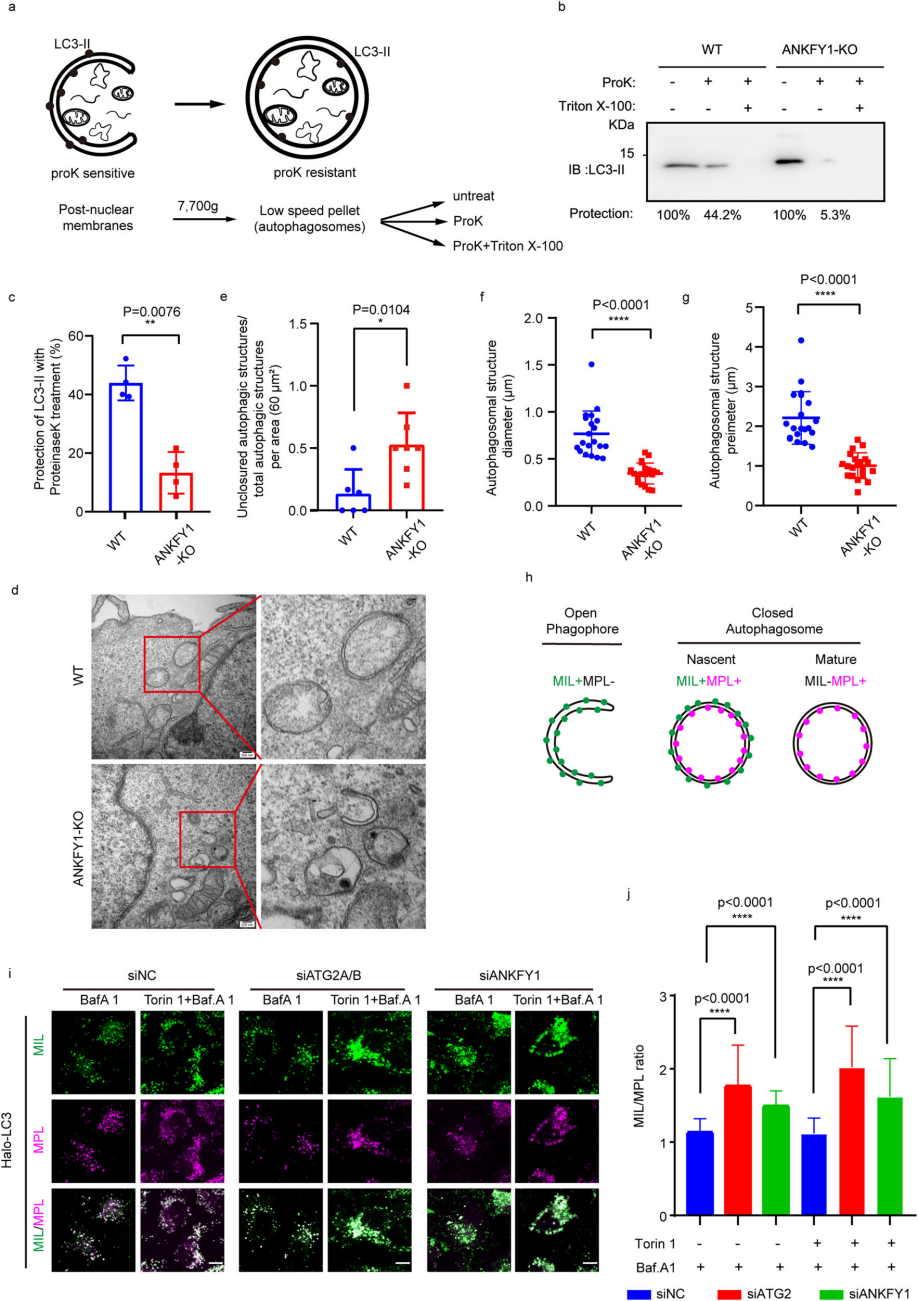
Depletion of ANKFY1 leads to defective autophagosome growth. **a** Schematic diagram outlining proteinase protection assay. **b** WT and ANKF1 KO U_2_OS cells were cultured in regular DMEM. Cell lysates were collected after treatment with 200 nM Torin 1 and 100 nM BafA1 for 2 h. The subfractions were separated by gradient centrifugation and then treated with 5 μg/mL proteinase K (ProK) with or without 0.5% Triton X-100. **c** Quantification of the results in **b**. Statistical significance was calculated by *t*-test. *P ≤ 0.05. **d** WT or ANKFY1 KO U_2_OS cells were treated with 200 nM Torin 1 for 2 h. Cells were fixed and imaged with transmission electron microscope. Scale bars, 200 nm. **e** Quantification of the ratio of unclosed autophagic structures to total autophagic structures per imaging area (60 μm^2^) in **d**. Data are mean ± SD; *n* = 6 imaging areas (WT) and 7 imaging areas (ANKFY1 KO). **f**, **g** Quantification of the results in **d**. Data are mean ± SD for 20 autophagosomes. ****P ≤ 0.0001. **h** Three populations of HT-LC3-II structures in the HT-LC3 autophagosome completion assay: MIL^+^MPL^−^, representing phagophores; MIL^+^MPL^+^, representing nascent autophagosomes; MIL^−^MPL^+^, representing mature autophagosomes, amphisomes, and autolysosomes. **i** HaloTag-LC3 (HT-LC3)-expressing U_2_OS cells were transfected with siNC, siATG2A/B or siANKFY1 for 48 h. Then cells were treated with or without 200 nM Torin 1 for 3 h in the presence of 100 nM BafA1 for 2 h and subjected to the HT-LC3 autophagosome completion assay followed by confocal microscopy. Scale bars, 20 μm. **j** MIL/MPL ratio for cells treated with or without Torin 1 in the presence of BafA1 was calculated from 50 cells in **i**. Statistical significance was determined by two-tailed *t*-test. *P*-values are listed.

As ATG2 depletion caused accumulation of unclosed autophagosome-related membranes^20^, we also assessed whether the depletion of ANKFY1 would cause the similar phenotype. We analyzed the autophagic membrane structures in ANKFY1 KO U_2_OS cells by differential centrifugation. Cells were homogenized and the post-nuclear supernatant (PNS) was fractionated by differential centrifugation. The membrane-bound LC3-II was recovered in the low-speed pellet (LSP) fraction. Part of LC3-II is protected by closed autophagosome from proteinase K if autophagosome formation is normal (Fig. 3a). In both WT and ANKFY1 KO U_2_OS cells, LC3-II was sensitive to proteinase K in the presence of Triton X-100, suggesting that the proteinase K worked well (Fig. 3b). Without Triton X-100, LC3-II was partially degraded by proteinase K in WT cells, but almost fully degraded in ANKFY1 KO cells treated with proteinase K (Fig. 3b, c). These data suggest that complete closure of the autophagosome was impaired in the absence of ANKFY1. Next, to better understand the nature of autophagosome-related structures upon ANKFY1 depletion, ANKFY1 WT and KO U_2_OS cells with Torin 1 treatment were observed under transmission electron microscope. We observed accumulated unclosed autophagosome-related structures in ANKFY1 KO cells (Fig. 3d, e). Remarkably, knockout of ANKFY1 led to smaller autophagosome and autophagosome-like structures, as evidenced by smaller diameter (Fig. 3f) and perimeter (Fig. 3g) of these structures.

Additionally, we used the HaloTag-LC3 (HT-LC3) autophagosome completion assay to further examine whether ANKFY1 knockout would lead to more unclosed autophagosome-related structures in cells. This assay was able to specifically detect phagophores, nascent autophagosomes, and mature autophagic structures by sequentially labeling cytosol-accessible and autophagosome-sequestered HT-LC3 by using fluorescently tagged membrane-impermeable (MIL) and membrane-permeable (MPL) HaloTag ligands, respectively (Fig. 3h)^20,21^. We generated U_2_OS cells stably expressing HT-LC3 for the autophagosome completion assay. The cells were transfected with the indicated siRNA and siATG2A/B was used as positive control. Cells were subjected to treatment with either BafA1 or a combination of Torin 1 and BafA1, followed by permeabilization of the plasma membrane to release cytosolic HT-LC3-I and sequential labeling of HT-LC3-II with MIL and MPL. Notably, the MIL/MPL ratio in the presence of BafA1 in siATG2- or siANKFY1-transfected cells was significantly higher than that in siNC cells (Fig. 3i, j). These results highly suggest that depletion of either ANKFY1 or ATG2A/B delays autophagosome completion under both basal and Torin1-treated conditions. Taken together, depletion of ANKFY1 reduces phagophore growth and closure.

### ANKFY1 associates with ATG2A in close proximity with endosomes and phagophores upon autophagy stimulation

To better elucidate the role of ANKFY1, we proceeded to examine its subcellular distribution and its co-localization with ATG2A. It was reported before that ANKFY1 mainly localizes on endosomes^18^. We first examined whether this localization would change with Torin 1 treatment. We used Rab5-mCherry to label endosomes and we found that ANKFY1 co-localized with Rab5 in untreated condition, which is consistent with previous published results^18^ (Supplementary Fig. S2a, upper panel). Upon Torin 1 treatment, all the ANKFY1 puncta still overlapped well with Rab5 puncta (Supplementary Fig. S2a, lower panel). This suggests that ANKFY1 localized on endosomes in both untreated and autophagy-stimulated conditions.

**Fig. 4:**
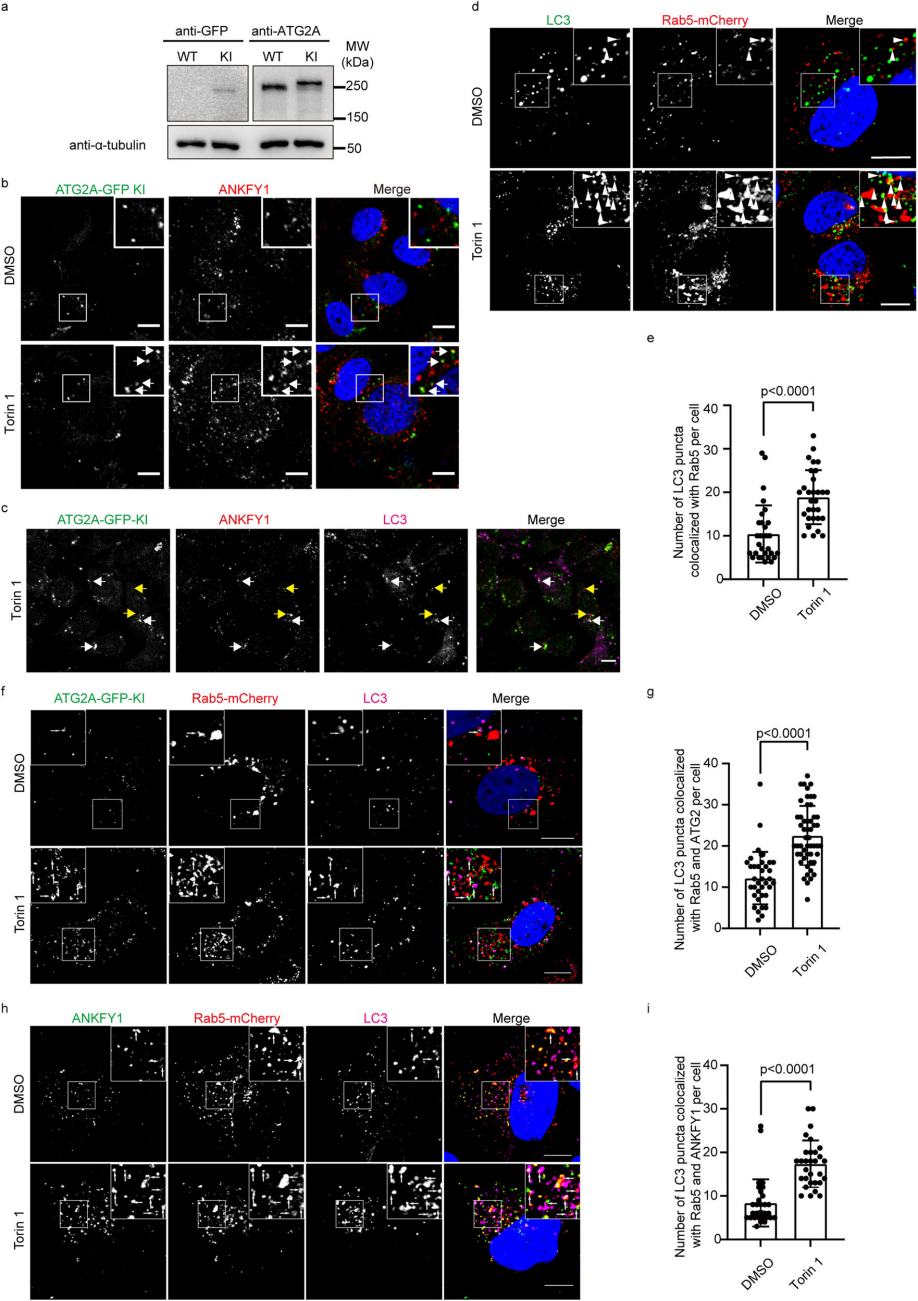
ANKFY1 associates with ATG2A in close proximity to endosomes and phagophores upon autophagy stimulation. **a** Expression level of ATG2A-GFP in ATG2A-GFP KI U_2_OS cells. Cell lysates were analyzed by immunoblotting with the indicated antibodies. **b** ATG2A-GFP KI U_2_OS were cultured in regular DMEM and treated with 200 nM Torin 1 for 3 h. Cells were fixed, stained with antibody against ANKFY1 and then imaged under confocal microscope. Scale bars, 10 μm. **c** ATG2A-GFP KI U_2_OS were cultured in regular DMEM and treated with 200 nM Torin 1 for 3 h. Cells were fixed, stained with the indicated antibodies and then imaged under confocal microscope. The white arrows indicate cases where ANKFY1 puncta are localized in close proximity to both ATG2A-GFP and LC3 puncta. The yellow arrows indicate cases where ANKFY1 puncta are localized in close proximity to LC3 puncta but not ATG2A puncta. Scale bars, 10 μm. **d** U_2_OS were cultured in regular DMEM and transfected with Rab5-mCherry, and then treated with 200 nM Torin 1 for 3 h. Cells were fixed, stained with antibody against LC3 and then imaged under confocal microscope. Scale bars, 10 μm. **e** Quantification of number of LC3 puncta in close proximity to Rab5-mCherry puncta per cell in **d**. Data are mean ± SEM from 30 cells. *P-*value is indicated (two-tailed paired *t*-test). **f** ATG2A-GFP KI U_2_OS were cultured in regular DMEM and transfected with Rab5-mCherry, and then treated with 200 nM Torin 1 for 3 h. Cells were fixed, stained with antibodies against GFP and LC3, and then imaged under confocal microscope. Scale bars, 10 μm. **g** Quantification of number of LC3 puncta in close proximity to Rab5-mCherry and ATG2A-GFP puncta per cell in **f**. Data are mean ± SEM from 36 cells (DMSO) and 48 cells (Torin 1). *P-*value is indicated (two-tailed paired *t*-test). **h** U_2_OS were cultured in regular DMEM and transfected with Rab5-mCherry, and then treated with 200 nM Torin 1 for 3 h. Cells were fixed, stained with antibodies against LC3 and ANKFY1, and then imaged under confocal microscope. Scale bars, 10 μm. **i** Quantification of number of LC3 puncta in close proximity to Rab5-mCherry and ANKFY1 puncta per cell in **h**. Data are mean ± SEM from 30 cells. *P-*value is indicated (two-tailed paired *t*-test).

Next, we investigated the co-localization between ANKFY1 and ATG2. To better visualize ATG2A subcellular localization, we generated ATG2A-GFP single copy knock-in (KI) U_2_OS cells as commercially available antibody against ATG2A was not effective in immunofluorescence and the overexpression of ATG2A might cause artificial localization of ATG2A on lipid droplets^11^. The expression level of ATG2A-GFP is comparable to that of the endogenous ATG2A by western blot (Fig. 4a). Without Torin 1 treatment, we barely observed any co-localization between ANKFY1 and ATG2A-GFP (Fig. 4b, upper panel); however, with Torin 1 treatment, ANKFY1 was found to co-localize with ATG2A-GFP markedly (Fig. 4b, lower panel, white arrows). As ATG2A was reported to locate on phagophores, we wondered whether ANKFY1 can also co-localize with phagophore markers upon autophagy induction. The results indicate that a portion of ANKFY1 puncta is in close proximity to both ATG2A puncta and LC3 puncta (Fig. 4c, white arrows).

Observing the partial overlap between endosome-localized ANKFY1 and phagophore-localized ATG2A during autophagy stimulation, we were intrigued by the possibility of endosomes and phagophores being in close proximity during autophagy. Interestingly, we found that under Torin 1 treatment, an elevated co-localization between endosomal marker Rab5-mCherry and phagophore marker LC3 was observed (Fig. 4d, e). Additionally, a three-color overlay of ATG2A-GFP, Rab5-mCherry, and LC3 showed the increased co-localization following Torin 1 treatment (Fig. 4f, g), indicating a substantial presence of ATG2A in close proximity to both phagophores and endosomes. Similarly, the three-color overlay of ANKFY1, Rab5-mCherry, and LC3 also showed an increased co-localization under Torin 1 treatment (Fig. 4h, i), suggesting that a significant pool of ANKFY1 are in close proximity to both phagophores and endosomes during autophagy stimulation.

These data collectively suggest that, under autophagy induction, a population of ANKFY1 associates with ATG2A in the vicinity of both phagophores and endosomes.

### ANKFY1 enhances lipid transfer by ATG2A in vitro

Considering the stimulated interaction and co-localization observed between ANKFY1 and ATG2A during autophagy induction, along with the similar effects caused by ANKFY1 depletion and ATG2 depletion, we questioned whether ANKFY1 collaborates with ATG2A in lipid transfer. The lipid transfer activity of ATG2A requires its intrinsic hydrophobic tunnel and membrane-anchoring proteins since ATG2A per se exhibits weak membrane binding. Firstly we purified ATG2A from mammalian cells and examined its lipid transfer activity in an in vitro system with an artificial membrane tether which has been described previously^11^. In this system, the lipid transfer activity was indicated by fluorescence resonance energy transfer (FRET) when a pair of self-quenched fluorescence dyes move apart to release fluorescence signals due to lipid transfer. We reconstituted donor liposomes containing FRET pair (NBD-PE, Rhod-PE) and His-tagged protein-binding lipids (DGS-NTA), and acceptor liposomes containing PI(4,5)P2 but lacking fluorescent lipids or DGS-NTA. Purified Flag-ATG2A-His protein, purified Flag-ANKFY1 protein, and an artificial His-PH polypeptide tether was added in the reaction (Supplementary Fig. S3a, b). ATG2A protein, per se, has weak membrane binding activity (Supplementary Fig. S1c, d); therefore a C-terminal His tag was designed to attach ATG2A to the donor membrane through binding to DGS-NTA in donor liposomes due to the His-NTA interaction. This system also includes an artificial tether, His-PH, which pulls donor and acceptor liposomes to close proximity through binding DGS-NTA in the donor liposomes by its N-terminal His-tag at one end, and through binding PI(4,5)P2 in the acceptor liposomes by its C-terminal PH domain at the other end. As expected, we observed ATG2A transferred lipids between tethered liposomes as evidenced by the increased NBD signal (Supplementary Fig. S3c, red curve) as other group reported^11^. We also observed that ANKFY1 enhanced this lipid transfer efficiency (Supplementary Fig. S3c, blue curve). Besides, without ATG2A, ANKFY1 could not transfer lipids between tethered liposomes (Supplementary Fig. S3c, green curve), which excluded the possibility that ANKFY1 per se is a lipid transfer protein.

**Fig. 5:**
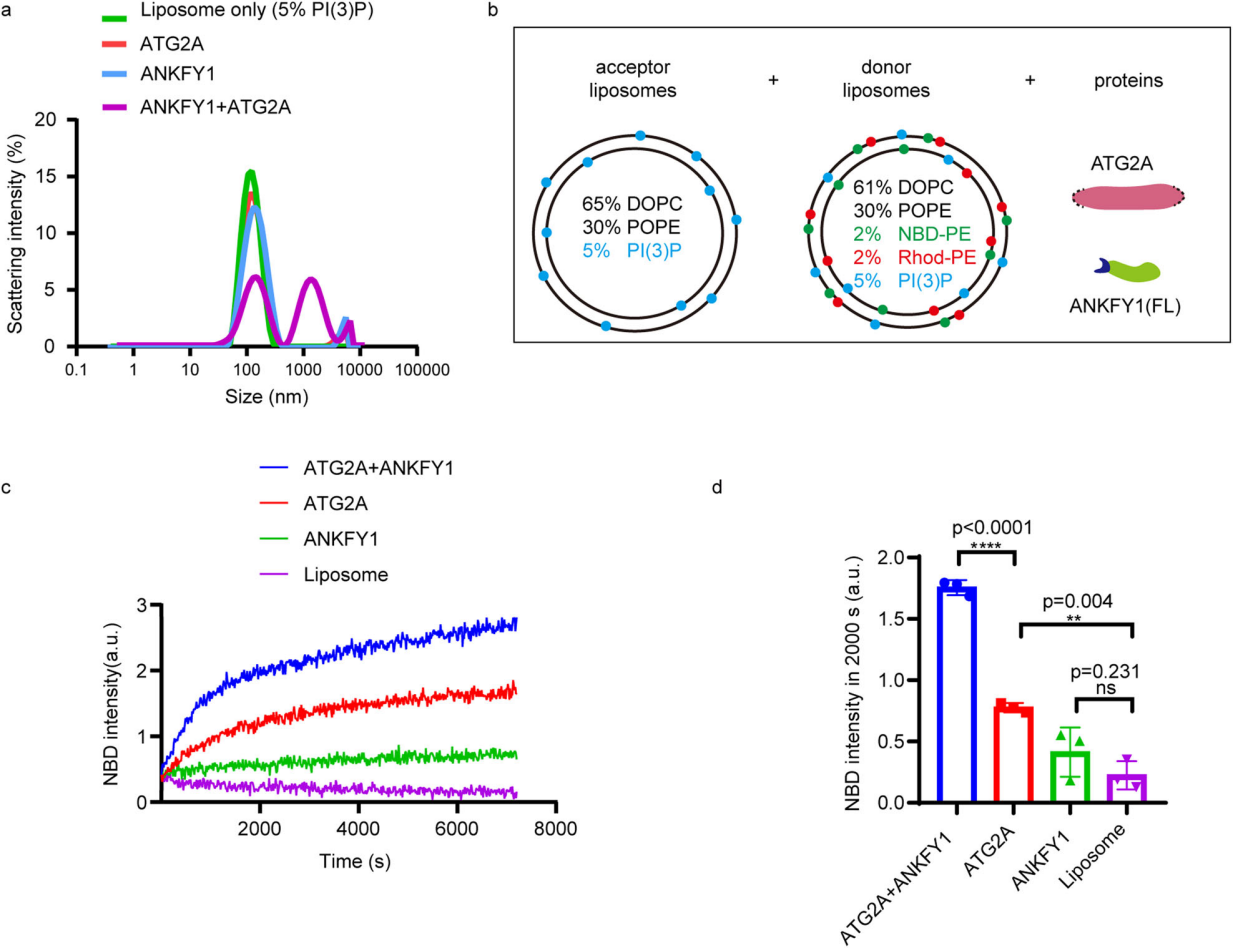
ANKFY1 promotes ATG2A-mediated lipid transfer in vitro. **a** Assessment of liposome size distribution by DLS. The 100 nm liposomes were reconstituted and incubated with the indicated proteins at 37 °C. **b** Schematic diagram of lipid transfer assay. Donor liposomes (61% DOPC, 30% POPE, 2% NBD-PE, 2% Rhod-PE, 5% PI3P) and acceptor liposomes (65% DOPC, 30% POPE, 5% PI3P) were mixed with ATG2A, ANKFY1 or both as indicated. **c** FRET-based lipid transfer assay as in **b**. The lipid transfer was measured by the increase of NBD fluorescence intensity. **d** Quantification of results in **c**. Bars represent averages of the NBD signal observed after 2000 s in three independent experiments. Error bars represent standard deviations. Data are mean ± SD. **P ≤ 0.01; ****P ≤ 0.0001; ns, not significant.

The major drawback of this in vitro system is the utilization of the artificial membrane tether rather than physiological membrane tethers. Given the interaction of ANKFY1 with PI3P and ATG2A, as well as its enhancement of the lipid transfer activity of ATG2A, we speculated whether ATG2A together with ANKFY1 could tether PI3P liposomes and transfer lipids without artificial linker. Using dynamic light scattering (DLS), we demonstrated that ATG2A together with ANKFY1 clustered liposomes containing 5% PI3P as evidenced by the increased particle size of a subset of liposomes, but neither ATG2A nor ANKFY1 alone could readily tether 100 nm liposomes containing 5% PI3P (Fig. 5a).

**Fig. 6:**
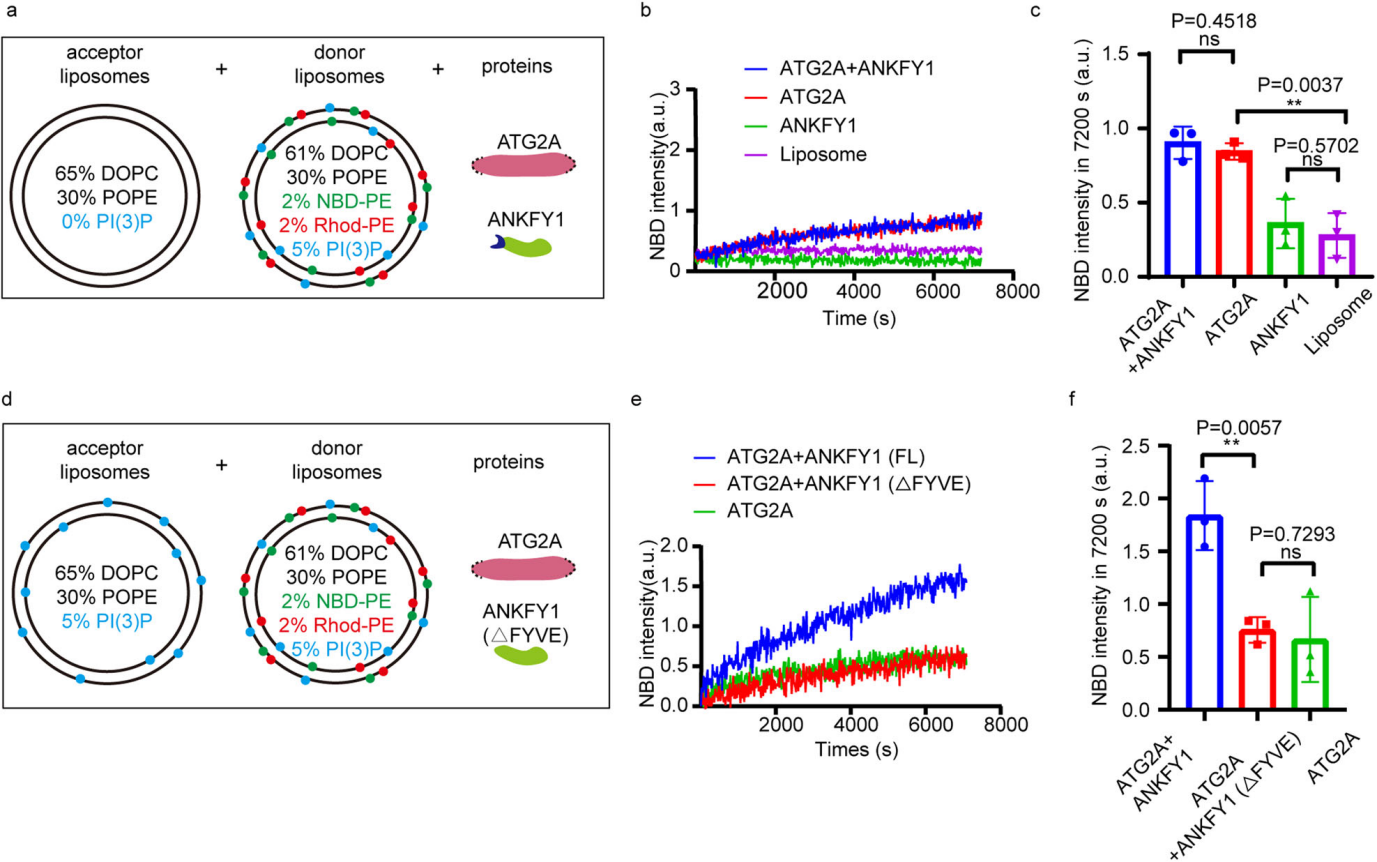
ANKFY1 binding to PI3P through FYVE domain is essential for ATG2A-mediated lipid transfer in vitro. **a** Schematic diagram of lipid transfer assay. Donor liposomes (61% DOPC, 30% POPE, 2% NBD-PE, 2% Rhod-PE, 5% PI3P) and acceptor liposomes (70% DOPC, 30% POPE) were mixed with ATG2A, ANKFY1 or both as indicated. **b** FRET-based lipid transfer assay as in **a**. The lipid transfer was measured by the increase of NBD fluorescence intensity. **c** Quantification of results in **b**. Bars represent averages of the NBD signal observed after 7200 s in three independent experiments. Error bars represent standard deviations. Data are mean ± SD. **P ≤ 0.01; ns, not significant. **d** Schematic diagram of lipid transfer assay. Donor liposomes (61% DOPC, 30% POPE, 2% NBD-PE, 2% Rhod-PE, 5% PI3P) and acceptor liposomes (65% DOPC, 30% POPE, 5% PI3P) were mixed with ATG2A and different constructs of ANKFY1 as indicated. **e** FRET-based lipid transfer assay as in **d**. The lipid transfer was measured by the increase of NBD fluorescence intensity. **f** Quantification of results in **e**. Bars represent averages of the NBD signal observed after 7200 s in three independent experiments. Error bars represent standard deviations. Data are mean ± SD. **P ≤ 0.01; ns, not significant.

Furthermore, we examined whether we can reconstitute lipid transfer activity of ATG2A and ANKFY1 skipping any artificial tether in the in vitro system. We removed the His-PH polypeptide away from the lipid transfer assay system. Besides, we took away the DGS-NTA from donor liposomes for ATG2A to bind artificially and PI(4,5)P2 from acceptor liposomes for the His-PH tether to bind. Instead, we added 5% PI3P to both donor liposomes and acceptor liposomes. In our modified lipid transfer assay, donor liposomes containing NBD-PE, Rhod-PE and 5% PI3P were mixed with acceptor liposomes containing 5% PI3P, and then ATG2A and ANKFY1 were added as indicated (Fig. 5b). The results showed that ATG2A can transfer lipids weakly in the absence of either artificial tether or the artificial liposomes anchor, DGS-NTA (Fig. 5c, d, red). Besides, ANKFY1 alone did not transfer lipids (Fig. 5c, d, green). Remarkably, ANKFY1 doubled the lipid transfer efficiency of ATG2A alone (Fig. 5c, d, blue vs red). These findings indicate that ANKFY1 promotes ATG2A-mediated lipid transfer in vitro, which is potentially attributed to an augmented liposome tethering function facilitated by the co-presence of both ATG2A and ANKFY1. Based on our previous results, ATG2A binds PI3P-containing liposomes weakly (Supplementary Fig. S1c, d), but ANKFY1 possessing one FYVE domain binds PI3P-containing liposomes tightly (Fig. 1f, g). We speculated that ANKFY1 enhances membrane tethering ability of ATG2A through interaction with PI3P on liposomes via its FYVE domain, leading to the increased lipid transfer efficiency. To test this hypothesis, we removed PI3P from the acceptor liposomes in the lipid transfer assay (Fig. 6a), and the enhanced lipid transfer by ANKFY1 was not observed (Fig. 6b, c). Similarly, ANKFY1 with FYVE domain deletion could not promote ATG2A-mediated lipid transfer between donor liposomes and acceptor liposomes both containing 5% PI3P (Fig. 6d–f; Supplementary Fig. S4a). Deletion of the FYVE domain in ANKFY1 hindered its localization on PI3P-rich endosomes, as indicated by a significant reduction in ANKFY1 puncta co-localizing with EEA1 puncta following Torin 1 treatment (Supplementary Fig. S4b, c). These results indicate that the interaction between FYVE domain of ANKFY1 and PI3P plays a crucial role in facilitating ATG2A-mediated lipid transfer.

### Depletion of ATG2, ANKFY1 or UVRAG reduced PI3P on phagophores

Based on the findings from our previous experiments, we have established that: (1) ANKFY1 interacts with ATG2A upon autophagy stimulation; (2) ANKFY1 depletion phenocopies ATG2 depletion in the regulation of autophagy flux and autophagosome expansion; (3) ANKFY1 associates with ATG2A in close proximity to both endosomes and phagophores during autophagy stimulation; and (4) ANKFY1 enhances lipid transfer by ATG2A in vitro. Therefore, we speculated that ANKFY1 assists in anchoring of ATG2 onto endosomes, facilitating the transfer of lipids from endosomes to phagophores. Given the known abundance of PI3P on endosomes, we tested whether the PI3P abundance on phagophores can be affected by PI3P production on endosomes and ATG2A-ANKFY1-mediated lipid transfer.

**Fig. 7:**
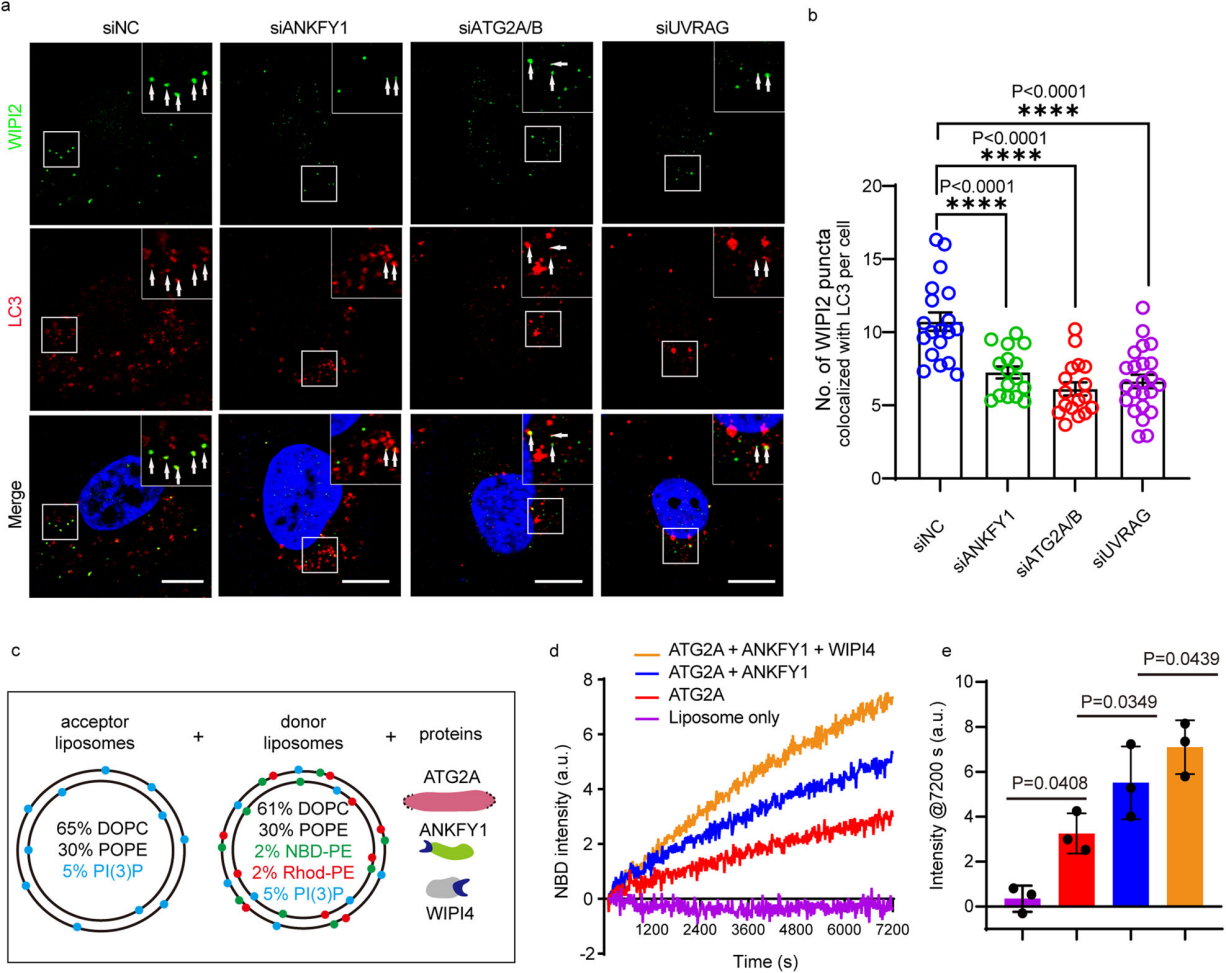
ATG2A-ANKFY1 mediate lipid transfer from endosomes to phagophores. **a** U_2_OS cells were transfected with non-targeting control siRNA or indicated siRNA and then cultured in regular DMEM. 48 h after transfection, cells were treated with 200 nM Torin 1 for 3 h, fixed and stained with DAPI and antibodies against WIPI2 and LC3. Cells were imaged under confocal microscope. Scale bars, 10 μm. **b** Quantification of the results in **a**. Data are mean ± SEM. *n* = 22 images containing 176 cells (siNC), 12 images containing 136 cells (siANKFY1), 18 images containing 246 cells (siATG2A/B), or 26 images containing 356 cells (siUVRAG) from two independent experiments. *P*-values are listed. **c** Schematic diagram of lipid transfer assay. Donor liposomes (61% DOPC, 30% POPE, 2% NBD-PE, 2% Rhod-PE, 5% PI3P) and acceptor liposomes (65% DOPC, 30% POPE, 5% PI3P) were mixed with ATG2A, ANKFY1 and WIPI4 as indicated. **d** FRET-based lipid transfer assay as in **c**. The lipid transfer was measured by the increase of NBD fluorescence intensity. **e** Quantification of results in **d**. Bars represent averages of the NBD signal observed after 7200 s in three independent experiments. Error bars represent standard deviations. Data are mean ± SD. **P ≤ 0.01; ns, not significant.

In U_2_OS WT cells we observed a subset of WIPI2 puncta stained by endogenous WIPI2 antibody co-localizing with LC3 puncta stained by endogenous LC3 antibody (Fig. 7a). We defined these WIPI2 puncta co-localizing with LC3 puncta as PI3P puncta on phagophores. As is widely known, PI3P production on endosomes is catalyzed by the PI3KC3C2 complex, whereas PI3P generation on phagophores is facilitated by the PI3KC3C1 complex. Following the depletion of UVRAG (Supplementary Fig. S5a), an endosome-localized subunit of the PI3KC3C2 complex, we observed a decrease in the number of WIPI2 puncta co-localizing with LC3 puncta, aligning with expectations (Fig. 7a, b). This suggests that PI3P on endosomes might serve as a source for PI3P on phagophores. Besides, with depletion of ATG2A and ATG2B (Supplementary Fig. S5a), the number of PI3P puncta on phagophores decreased (Fig. 7a, b), which indicates that a part of PI3P lipids on phagophores came from other membrane sources delivered by ATG2A/B, rather than being fully dependent on the production by the PI3KC3C1 complex on phagophore. Furthermore, we noted that ANKFY1 knockdown resulted in a decrease in PI3P content on phagophores to a similar extent to ATG2A/B knockdown or UVRAG knockdown (Fig. 7a, b). These data suggest that ATG2 together with ANKFY1 might transfer PI3P lipids from endosomes to phagophores upon autophagy stimulation.

One thing worth mentioning is that, in our simplified in vitro lipid transfer system (Fig. 5b–d), we only included ANKFY1 and ATG2A, and we anticipated that under physiological settings, there should be other membrane-anchoring proteins, such as WIPI4 on the autophagosome membrane. We explored the collaborative impact of WIPI4 and ANKFY1 on the lipid transfer activity of ATG2A in vitro. We purified recombinant WIPI4 protein from mammalian cells (Supplementary Fig. S6a). Donor liposomes containing 5% PI3P and acceptor liposomes with 5% PI3P were mixed with either ATG2A alone, ATG2A and ANKFY1, or ATG2A, ANKFY1 and WIPI4 together (Fig. 7c). Remarkably, the presence of both ANKFY1 and WIPI4 led to a significant enhancement in ATG2A-mediated lipid transfer, surpassing the enhancement achieved by ANKFY1 alone (Fig. 7d, e, yellow vs blue). This observation underscores that the synergistic collaboration between WIPI4 and ANKFY1 can further amplify the lipid transfer activity of ATG2A.

In a previous study, it was revealed that amino acids 1358–1404 of ATG2A directly bind to WIPI4 through XLS-MS analysis^12^. The other study by the same group proposed that the CAD tip of ATG2A is anchored to the phagophore via WIPI4, while the N-tip makes contact with the ER membrane^13^. Our immunoprecipitation results aligned with these findings, confirming the interaction between ATG2A-C (1113–1938) and WIPI4 as previously reported (Supplementary Fig. S7a). Consequently, we hypothesize that the other tip (1–345) of ATG2A may interact with ANKFY1 on the endosome. Our immunoprecipitation results indeed demonstrated the interaction between ANKFY1 and ATG2A (1–345) (Supplementary Fig. S7a, b). Notably, the expression of ANKFY1 did not disrupt the interaction between ATG2A-C (1113–1938) and WIPI4 (Supplementary Fig. S7a). Similarly, the expression of WIPI4 did not disrupt the interaction between ATG2A-N (1–345) and ANKFY1 (Supplementary Fig. S7b). These results suggest that the ATG2A could simultaneously interact with ANKFY1 and WIPI4 through different terminus (Supplementary Fig. S7a).

## Discussion

ATG2 is recently recognized as a lipid transfer protein for autophagosome formation. In this study, we identified an ATG2A-binding protein ANKFY1 that can facilitate lipid transfer by ATG2A from endosomes to phagophores. This notion is supported by the following evidences: (1) ANKFY1 was identified by unbiased MS analysis of tandem affinity purification of ATG2A under autophagy stimulation condition; (2) autophagy-inducing conditions enhanced the interaction between ANKFY1 and ATG2A; (3) Depletion of ANKFY1 reduced autophagy flux and autophagosome growth, which phenocopied the depletion of ATG2A/B; (4) ANKFY1 and ATG2A co-localized in close proximity to endosomes and phagophores upon Torin 1 treatment; (5) ANKFY1 enhanced the ATG2A-mediated lipid transfer in vitro and PI3P binding of ANKFY1 via FYVE domain is essential for this; (6) PI3P content on phagophores was reduced when UVRAG, ATG2A/B or ANKFY1 was depleted.

The membrane source of autophagosomes is extensively studied but still under debate^7^. A model that has been generally accepted is that phagophore arises from some certain membrane precursors, which grow by either fusing with each other or receiving lipids from other membrane sources^7,22,23^, including ER-exit sites (ERES) and the ER–Golgi intermediate compartment (ERGIC)^24,25^, the Golgi apparatus^26^, mitochondria^27^, the plasma membrane^28^, early endosomes^29^ and recycling endosomes^30^. For endosomes as the lipid sources for autophagosome biogenesis, it was proposed that ATG9-positive vesicles were formed from endosomal membrane tubulation to transport lipids to the autophagosome formation sites upon autophagy stimulation. In our study, we proposed a new model in which ATG2A transfers lipids from endosomes to phagophores, and the endosome-localized ANKFY1 facilitates this process by stabilizing ATG2A anchoring on endosomes.

It is worth noting that, in ANKFY1 KO cells, the LC3-II accumulation upon Torin 1 treatment was further increased upon BafA1 treatment (Fig. 2f, lane 6 vs lane 8), suggesting that depletion of ANKFY1 does not cause a complete block in autophagy flux as double knockout of ATG2A/B reported by other group^10,11^. As membrane input from endosomes to the forming autophagosome is only one of several possible membrane sources, we do not expect the effect of ANKFY1 depletion to be as strong as that of a core autophagy protein depletion. Up to this point, we have successfully reconstituted an in vitro lipid transfer system comprising ATG2A, WIPI4, and ANKFY1. Nevertheless, additional factors, including ATG9, among others, might play a role in this process^31^. In the future, the inclusion of these essential players in the reconstituted system will be beneficial for gaining a more comprehensive understanding of how ATG2 mediates lipid transfer for phagophore growth.

In summary, here we reported that ANKFY1 facilitates lipid transfer by ATG2A from endosomes to autophagosome formation sites. These findings shed a light on our understanding of lipid transfer model for phagophore expansion, where endosomes can also donate membranes to phagophores via lipid transfer.

## Materials and methods

### Antibodies and reagents

Antibodies used in this study were as follows: anti-ANKFY1 (Santa Cruz, sc-393353), anti-ATG2A (Cell Signaling, 15011S), anti-Flag (Sigma-Aldrich, F1804), anti-GFP (Abcam, ab290), anti-LC3 (for western blot, Sigma-Aldrich, L7543; for immunofluorescence, MBL, PM036 or M152-3), anti-SQSTM1 (Novusbio, H00008878-M01), anti-α-tubulin (Proteintech, Biotin-66031), anti-Cathepsin B (Cell Signaling, 31718T), anti-ATG9A (Cell Signaling, 13509S), anti-EEA1 (Abcam, ab109110), anti-WIPI2 (Abcam, ab105459), anti-mCherry (ABclonal, AE002), Alexa Fluor 488-conjugated anti-mouse IgG (Jackson, 115-545-166), Alexa Fluor 594-conjugated anti-mouse IgG (Jackson, 115-585-166) and anti-rabbit IgG (Jackson, 111-585-144), IRDye 680RD Goat anti-rabbit (H + L) (Licor, 926-68071).

Reagents used in this study were as follows: Torin 1 (UBP Bio, F6101), Bafilomycin A1 (Selleckchem, S1413), lipid strips (Echelon Biosciences, P-6001), proteinase K (Roche, 3115879001), LysoTracker (Enzo, ENZ-51005), anti-Flag M2 Affinity Gel (Sigma-Aldrich, A2220), DAPI (Thermo Fisher, P36962), Fos-Choline-12 (Anatrace, F308), 3× Flag peptide lyophilized powder (Sigma-Aldrich, F4799), protease inhibitor cocktail (APExBIO, K1007), Lipofectamine 3000 (Thermo Fisher Scientific, L3000-015), TMR-conjugated Halo ligand (Promega, G825A), HaloTag Alexa Fluor 488 ligand (Promega, G1001). Lipids were purchased from Avanti Polar Lipids: DOPC (850357), POPE (850757), NBD-PE (810144), Rhod-PE (810158), PI3P (850187).

### Oligonucleotides

For siRNA-mediated gene knockdown, siRNA duplexes were purchased from GenePharma. The targeting sequences were:

ANKFY1-1, 5′-GGACUUCAUUUGAUGAGAA-3′,

ANKFY1-2, 5′-GAAACUAGCAAAUCGGUUU-3′,

ANKFY1-3, 5′-GCAUUGUCGGUGAUUCCAA-3′,

ATG2A-1, 5′-GCUACUUGCUGCACCACUA-3′,

ATG2A-2, 5′- GCUCCGACCUACAUGGUAU-3′,

ATG2A-3, 5′-CCCUGGACAGUGAUGAGUU-3′,

ATG2B-1, 5′-GCUCUUUACUGCAGGAUAA-3′,

ATG2B-2, 5′-GGGUGUAUCUUCAGAGCAA-3′,

ATG2B-3, 5′-GCAGUAGCUUUCUUUACUU-3′,

UVRAG-1, 5′-UCACUUGUGUAGUACUGAA-3′,

UVRAG-2, 5′-GCCCGGAACAUUGUUAAUA-3′,

Non-targeting control, 5′-UUCUUCGAACGUGUCACGU-3′.

For establishment of ANKFY1 KO U_2_OS cell line by CRISPR/Cas9, the sgRNA used is: 5′-CCATCGTGGCAGACCTCTACGAG-3′.

### Plasmid construction

Genes encoding Flag-tagged human ANKFY1 and ATG2A were cloned into pCDNA4.0-TO vector for eukaryotic expression. The construct corresponding to ANKFY1-ΔFYVE was made from Flag-ANKFY1-full-length (FL) by deleting residues 1104–1169. *ANKFY1* was also subcloned into pLV-GFP vector. *RAB5* was cloned into pLV-mCherry vector. The gene encoding Halo-LC3 was cloned into pLV-puro vector.

### Cell culture and transfection

HEK293T and U_2_OS cells were cultured in DMEM supplemented with 10% FBS and 1% penicillin-streptomycin solution. Cell transfection was performed using Lipofectamine 3000 (Invitrogen, L3000015) or PEI (Polysciences, 24765-1) according to protocols provided by the manufacturers. siRNAs were transfected into U_2_OS cells using Lipofectamine RNAiMAX (Invitrogen, 13778-150), according to the manufacturer’s protocol.

### Generation of ANKFY1 KO U_2_OS cells

The ANKFY1 KO U_2_OS cells were generated by CRISPR/Cas9-mediated genome editing. The sgRNAs were cloned into PX330 (#42230) vectors and U_2_OS cells were transfected by the resulting PX330 encoding Cas9 and gRNA using viral infection. 48 h later, cells were treated with 1 μg/mL puromycin for 3–5 days. Surviving cells were then isolated as single cell clones by limiting dilution and the KO clones were identified by western blot.

### Generation of ATG2A-GFP KI U_2_OS cells

The ATG2A-GFP KI U_2_OS cells were generated by CRISPR/Cas9 editing strategy. The sgRNA targeting sequence was 5′-AAGACTGAGCCTGGGGTGCC-3′. Briefly, the sgRNA was ligated into the *Bbs*I sites of PX330 vector. The template plasmids were constructed using pCRIS-PITChv2 vector (#63672), with the insertion of a left homologous arm (840 bp genomic DNA sequence upstream of the TAA region of ATG2A) into *Eco*RV/*Bam*HI sites, and a right homologous arm (790 bp genomic DNA sequence downstream of TAA region of ATG2A) into *Xba*I/*Nhe*I sites. The sgRNA and the template plasmids were co-transfected into cells. ATG2A-GFP KI cells were selected by puromycin and verified by western blotting. Finally, the single clone was obtained and identified by western blot and imaging.

### Autophagy flux assay

For autophagy induction, cells were incubated with 200 nM Torin 1 in U_2_OS complete medium for 3 h. To block autophagy, 100 nM BafA1 was added to U_2_OS complete medium and incubated for 3 h. Then, samples were collected for western blotting.

### Halo-LC3 turnover assay

WT and ANKFY1 KO U_2_OS cells were infected with lentivirus expressing Halo-LC3B for 24 h. Cells were incubated in complete culture medium with 100 nM TMR-conjugated Halo ligand for 30 min, and then treated with Torin 1 (200 nM, 3 h), BafA1 (100 nM, 3 h), or combination of both treatments. After 3 h, the cells were then collected for protein extraction for in-gel fluorescence imaging. For each sample, proteins were separated by SDS-PAGE, and the gel was immediately visualized with ChemiDoc Imaging system (Bio-Rad) after SDS-PAGE.

### Tandem affinity purification

The coding sequence of ATG2A was inserted between *Eco*RV and *Not*I cleavage sites in pCDNA5-TO vector to generate the open reading frame of ZZ-TEV-Flag-tagged bait protein. We generated a 293FT cell line that can stably express ZZ-TEV-Flag-bait protein by Lipofectamine 3000 transfection. The expression level of bait protein is controlled to endogenous level by addition of 1.5 ng/mL doxycycline. The tandem affinity purification of tagged bait protein was performed in a physiological buffer condition (20 mM Tris-HCl, pH 7.5, 150 mM NaCl, 0.5% NP-40, 1 mM EDTA, protease inhibitor mixture). The eluted protein mixtures were subjected to SDS-PAGE and further analyzed by MS. Procedures with detailed information was described before^32^.

### Immunofluorescence staining

Cells were grown on coverslips and transfected with the desired plasmids. After 48 h, cells were fixed using ice-cold 4% paraformaldehyde (PFA) for 30 min. Cells were then washed 3 times with PBS and blocked with PBS containing 5% FBS and 0.1% Saponin at 25 °C for 30 min. Cells were incubated with primary antibodies at 4 °C overnight, washed 3 times with PBS and then incubated with appropriate secondary antibodies for 1 h at 25 °C. Slides were examined under a laser scanning confocal microscope (Olympus FV3000). In experiments in Fig. 4c, f, h, GFP fluorescence or Alex Fluor 488 fluorescence was imaged using a 488 nm excitation and a 500–540 nm emission filter, mCherry fluorescence or Alex Fluor 561 fluorescence was imaged using a 561 nm excitation and a 570–620 nm emission filter, and Alex Fluor 647 fluorescence was imaged using a 640 nm excitation and a 650–670 nm emission filter.

### HaloTag-LC3 autophagosome completion assay

U_2_OS cells expressing HaloTag-LC3 were incubated in complete medium containing MPL at 37 °C for 15 min. Alternatively, cells were permeabilized with 20 μM digitonin at 37 °C for 2 min and incubated with MIL at 37 °C for 15 min. Cells were then fixed in 4% PFA for 5 min, washed three times in PBS, and incubated with MPL for 30 min. After three additional washes in PBS, cells were analyzed by confocal microscopy.

### Electron microscopy analysis

For transmission electron microscopy, cells were fixed in 2.5% glutaraldehyde in 0.1 M sodium cacodylate buffer (pH 7.4), followed by 1% osmium tetroxide in 0.1 M sodium cacodylate buffer (pH 7.2) for 2 h. Samples were blocked with 0.5% aqueous uranyl acetate overnight and subjected to low-temperature dehydration and infiltration with a graded series of Epon/Araldite, which was followed by the embedment in 100% Epon/Araldite. Thin sections (60 nm) were cut and stained with Reynalds lead citrate, and analyzed with a HITACHI H-7650 transmission electron microscope.

### Immunoprecipitation and immunoblotting

HEK293T cells were lysed using lysis buffer (20 mM Tris-HCl, pH 7.5, 150 mM NaCl, 0.5% NP-40, 1 mM EDTA and protease inhibitor cocktail) on ice. Whole-cell lysates were collected after centrifugation at 20,000× *g* at 4 °C for 30 min. Lysate was then incubated with antibody and IgG Affinity Gel or anti-Flag M2 Affinity Gel (Sigma), for 16 h at 4 °C. Beads were washed three times with lysis buffer and then were eluted with protein loading buffer or lysis buffer containing 0.25 µg/mL 3× Flag peptide. Elution supernatant was collected and immunoblotting was performed following standard procedures.

### Protein purification

Expression and purification of ATG2A and His-PH were described before^11^. The ANKFY1-FL and ANKFY1-ΔFYVE proteins were expressed and purified from 293S cells. Briefly, the human ANKFY1 or ANKFY1-ΔFYVE with a N-terminal Flag tag was cloned into pCDNA4-TO vector. Cells were harvested by centrifugation 72 h after transfection, resuspended in lysis buffer (20 mM HEPES, pH 7.4, 500 mM NaCl, 2 mM DTT, 1 mM EDTA and 1% Triton X-100) supplemented with protease inhibitors, and then were subjected to French press. The cell lysates were clarified via centrifugation at 21,000 rpm for 1 h. Clarified cell lysates were incubated with anti-Flag M2 resin (Sigma-Aldrich) at 4 °C overnight. Resin was subsequently washed with three column volumes of wash buffer (20 mM HEPES, pH 7.4, 500 mM NaCl, 1 mM TCEP, 1 mM EDTA and 0.1% FOS-CHOLINE-12) and the target protein was eluted with wash buffer containing 0.2 µg/mL 3× Flag peptide.

The WIPI4 protein was expressed and purified from 293S cells. Briefly, the human WIPI4 with a N-terminal Flag tag was cloned into pCDNA4-TO vector. Cells were harvested by centrifugation 72 h after transfection, resuspended in lysis buffer (20 mM HEPES, pH 7.4, 150 mM NaCl, 2 mM DTT, 1 mM EDTA) supplemented with protease inhibitors, and then were subjected to French press. The cell lysates were clarified via centrifugation at 21,000 rpm for 1 h. Clarified cell lysates were incubated with anti-Flag M2 resin (Sigma-Aldrich) at 4 °C overnight. Resin was subsequently washed with three column volumes of wash buffer (20 mM HEPES, pH 7.4, 300 mM NaCl, 1 mM TCEP, 1 mM EDTA) and the target protein was eluted with lysis buffer containing 100 µg/mL 3× Flag peptide.

### Proteinase protection assay

U_2_OS cells were grown in 15 cm dishes until > 90% confluence and treated with 200 nm Torin 1 and 100 nm BafA1 for 3 h. Cells were then washed with ice-cold PBS, centrifuged at 800× *g* for 5 min at 4 °C, resuspended in 500 μL homogenization buffer (10 mM HEPES, pH 7.4, 0.22 M mannitol, and 0.07 M sucrose) with EDTA-free proteinase inhibitor cocktail, and lysed with 27 G needle for 10 times. Lysates were first centrifuged at 300× *g* for 5 min in 4 °C. Postnuclear supernatants were further spun at 7700× *g* for 5 min at 4 °C. Pellets were saved as a LSP and resuspended in 50 μL homogenization buffer. For each proteinase protection assay, an equal protein amount of each sample was incubated alone, with 5 μg/mL proteinase K and 0.5% Triton X-100 on ice for 30 min. Samples were boiled for 10 min to stop the reactions and subjected to SDS-PAGE and immunoblotting.

### Co-flotation assay

Liposome co-floatation assay was performed as previously described^33^. Briefly, lipid compositions in the co-flotation assay are 63% DOPC, 30% POPE, 5% PI3P and 2% Rhod-PE. Lipid films were resuspended in buffer (50 mM HEPES, pH 8.0, 300 mM NaCl and 1 mM TCEP) and vortexed for 5 min. The re-suspended lipid films were frozen and thawed for 5 times, then extruded through 100 nm polycarbonate filter with an Avanti extruder for 21 times. Liposome solutions containing 1 mM lipids were incubated with 4.28 μM ANKFY1, 0.5 μM ATG2A or both proteins at 37 °C for 1 h. The liposomes and protein mixture were subjected to centrifugation on a histodenz density gradient (40%:35%:30%). Samples from the top to the bottom of the gradient were taken individually, and analyzed by SDS-PAGE, Coomassie blue staining and western blot.

### Protein–lipid overlay assay

The ANKFY1-Flag was purified from 293S cells as described above. Lipid strips were blocked in PBS with 5% BSA for 1 h at room temperature and then incubated with 0.5 μg/mL ANKFY1 in blocking buffer at 4 °C overnight. After 3 washes in PBS with 0.01% Tween-20, strips were immunoblotted with anti-Flag antibody.

### In vitro lipid transfer assay

Liposomes for lipid transfer assays were prepared as described above. The lipid compositions of donor liposomes and acceptor liposomes are labeled in the cartoon scheme in corresponding figures. All donor liposomes contained 2% NBD-PE and 2% Rhod-PE. 3.6 μM ANKFY1, 0.4 μM ATG2A or both proteins were mixed with 1 mM donor and acceptor liposomes in a cuvette and placed in a Spectrofluorometer (Varian Eclipse) at 37 °C. NBD fluorescence was recorded at an excitation wavelength of 460 nm and an emission wavelength of 538 nm for 7200 s. All data were base line corrected. At least three independent experiments were performed.

### Measuring the clustering activity of ATG2A and ANKFY1 by DLS

The clustering activity of ATG2A and ANKFY1 were measured by DLS using a DynaPro instrument (Malven, Zetasizer Nano series). For experiments with liposomes and proteins, the conditions and liposome compositions were similar to those of the liposomes used for lipid co-flotation assays unless specifically indicated. Thus, 3.6 μM ANKFY1 and 0.4 μM ATG2A or both proteins were mixed with liposomes (1 mM lipids) and incubated in a buffer containing 50 mM HEPES, pH 8.0, 300 mM NaCl and 1 mM TCEP at 37 °C for 1 h.

### Statistical analysis

Immunoblot signals and immunofluorescence images were processed and quantified using Fiji. Statistical analysis was carried out using GraphPad Prism 9. Data were evaluated by two-tailed unpaired Student’s *t*-test and represented as mean ± SD, with a *P-*value of < 0.05 being considered significant and a *P*-value of > 0.05 was considered statistically non-significant (ns).

### Supplementary information

Supplementary information
