# Supplementary material for: ANKFY1 bridges ATG2A-mediated lipid transfer from endosomes to phagophores
Source: Cell Discov. 2024 Apr 16;10:43. doi: 10.1038/s41421-024-00659-y (PMC11018839; doi:10.1038/s41421-024-00659-y)
Supplement: Supplementary file 1 — Supplementary information [file 41421_2024_659_MOESM1_ESM.pdf]

Supplementary Fig. S1

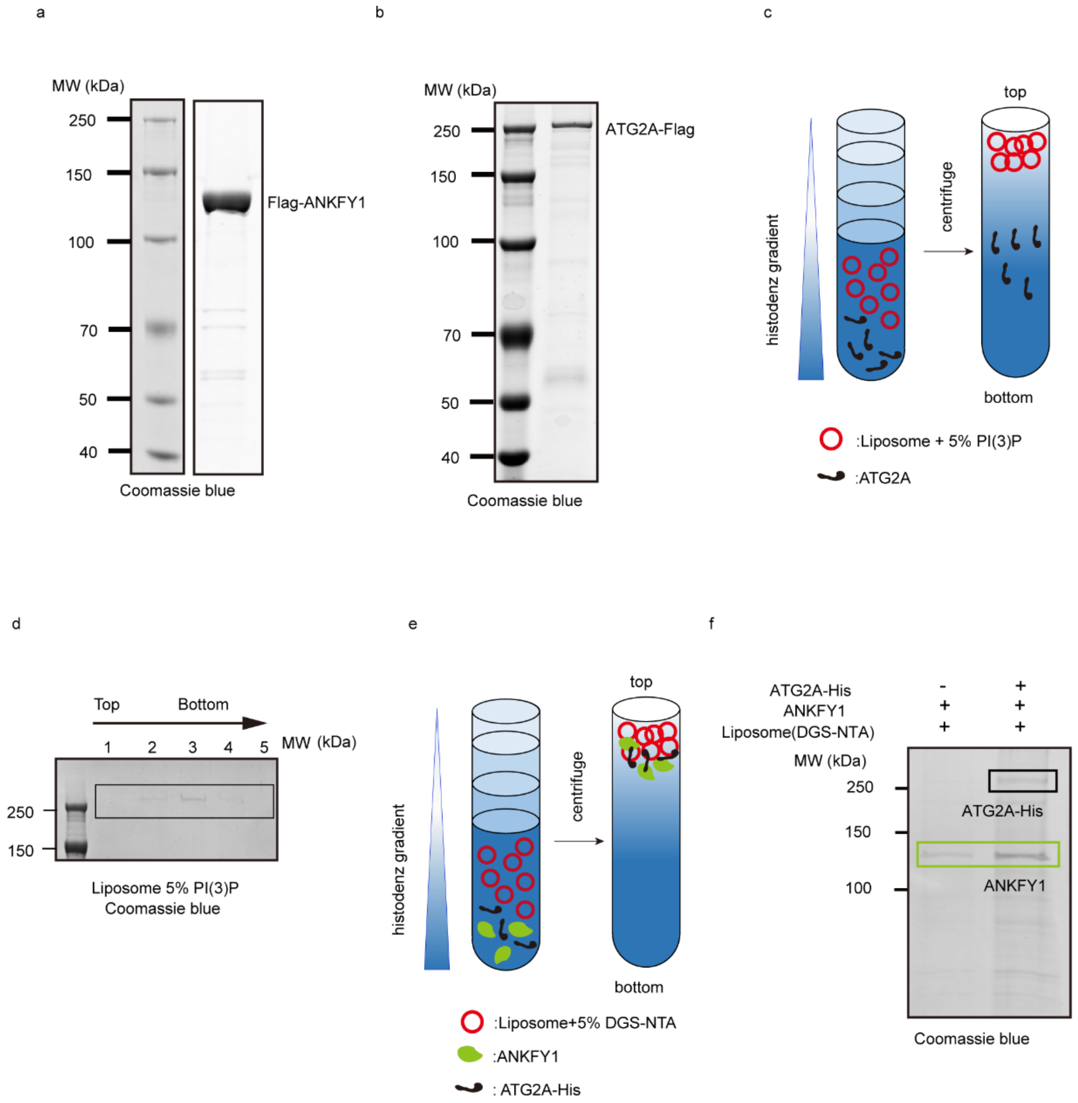

Supplementary Fig. S1: Detection of interaction between purified ANKFY1 and ATG2A.

(a) ANKFY1(FL) purified from HEK293S was analyzed by SDS-PAGE with Coomassie blue staining. **(b)** ATG2A purified from HEK293S was analyzed by SDS-PAGE with Coomassie blue staining. **(c)** Schematic diagram outlining of liposomes co-floatation assay. Liposomes (100 nm in size) containing 61% DOPC, 30% POPE, 4% RhoD-PE and 5% DGS-NTA were incubated with ATG2A-His and ANKFY1, then subject to floatation on a three-layer histodenz gradient (35%, 25%, and 0%). The top fraction was evaluated by SDS-PAGE. **(d)** The topmost fraction in **(c)** was analyzed by SDS-PAGE and Coomassie blue. **(e)** Schematic diagram outlining of liposomes co-floatation assay. Liposomes (100 nm in size) containing 61% DOPC, 30% POPE, 4% RhoD-PE and 5% DGS-NTA were incubated with ATG2A-His and ANKFY1, then subject to floatation on a three-layer histodenz gradient (35%, 25%, and 0%). The top fraction was evaluated by SDS-PAGE. **(f)** The topmost fraction in **(e)** was analyzed by SDS-PAGE and Coomassie blue.

Supplementary Fig. S2

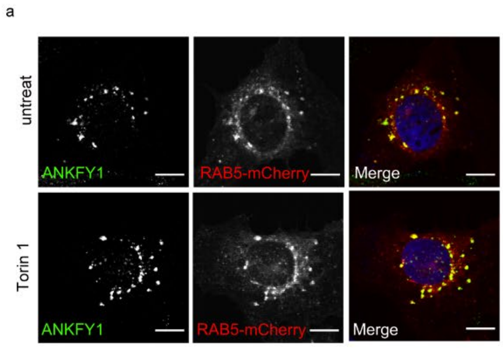

Supplementary Fig. S2: The localization of ANKFY1 on endosomes.

(a) U_2OS cells were cultured in regular DMEM and transfected with RAB5-mCherry, and then treated with or without 200 nM Torin 1 for 3 h. Cells were fixed and stained with endogenous ANKFY1 antibodies and then imaged under confocal microscope. Scale bar: 10 μm.

Supplementary Fig. S3:

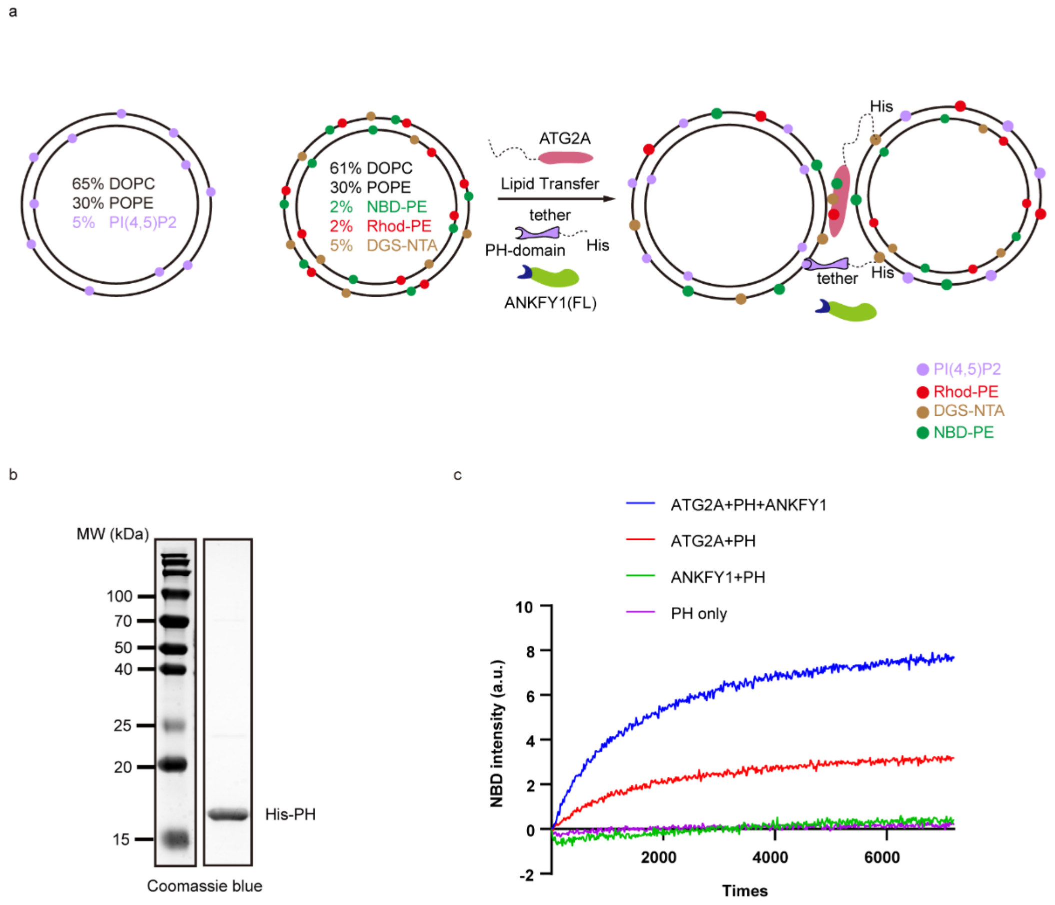

Supplementary Fig. S3: ATG2A transfers lipids between tethered liposomes *in vitro*. **(a)** Schematic diagram of Lipid transfer assay. Donor liposomes (61% DOPC, 30% POPE, 2% NBD-PE, 2% Rhod-PE, 5% DGS-NTA) and acceptor liposomes (65% DOPC, 30% POPE, 5% PI45P2) were tethered together by His-PH. Flag-ATG2A-His and ANKFY1 were added into the system by binding to the donor liposomes. The assay monitors the increase in NBD-PE fluorescence after lipid transfer from donor liposomes, where NBD fluorescence was quenched via Rhod-PE to acceptor liposomes. **(b)** His-PH purified from *E. coli* was analyzed by SDS-PAGE with Coomassie blue staining. **(c)** The ATG2A mediated lipid transfer was measured by the increasing of NBD fluorescence intensity in FRET-based lipid transfer assay as in **(a)**.

Supplementary Fig. S4

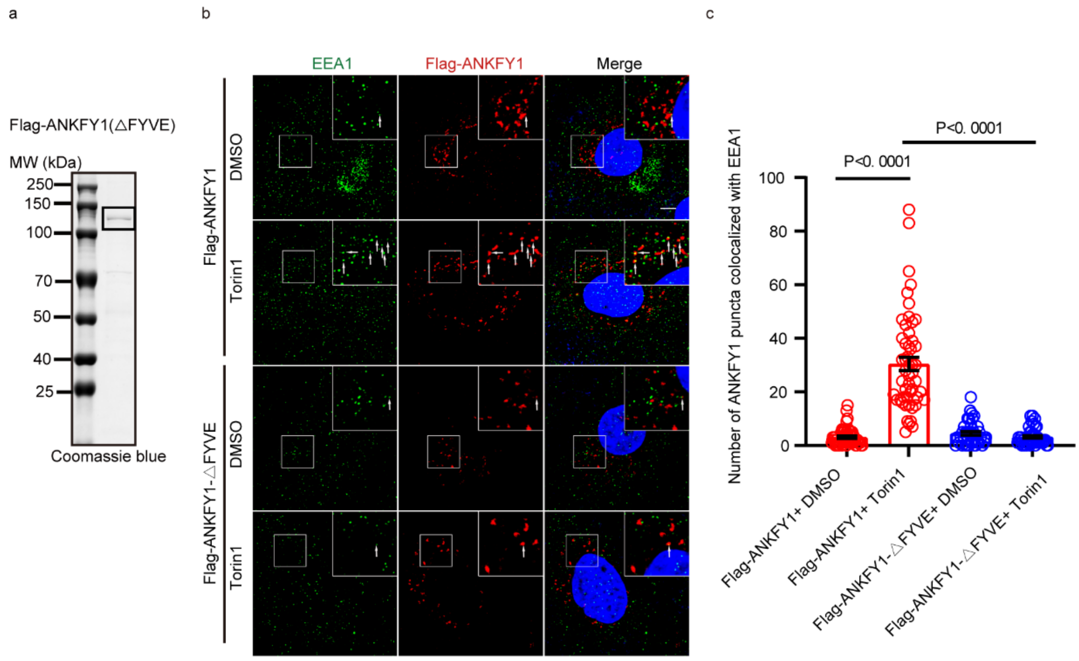

Supplementary Fig. S4: The importance of the FYVE domain in ANKFY1 localization on endosomes.

(a) ANKFY1(△ FYVE) purified from HEK293S was analyzed by SDS-PAGE with Coomassie blue staining. **(b)** The U_2OS cells was transfected with Flag-ANKFY1 or Flag-ANKFY1△FYVE. Cells were cultured in regular DMEM and treated with or without 200 nM Torin1 for 3 h. Cells were fixed and stained with endogenous antibodies against EEA1 and then imaged under confocal microscope. Scale bar 10 μm. **(c)** Quantification of ANKFY1 puncta colocalized with EEA1 per cell in **(b)**. Data are mean +/- SEM; n=52 (Flag-ANKFY1+DMSO), 52 (Flag-ANKFY1+Torin1), 38 (Flag-ANKFY1-△FYVE+DMSO), 41 (Flag-ANKFY1-△FYVE+Torin1). Significance was calculated by unpaired t test. P values are listed.

Supplementary Fig. S5

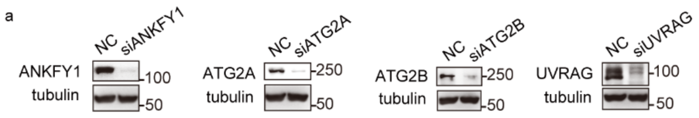

Supplementary Fig. S5: The knockdown efficiency of ANKFY1, ATG2A, ATG2B and UVRAG.

(a) The knockdown efficiency of ANKFY1, ATG2A, ATG2B and UVRAG in U_2OS in Figure 7a. Cell lysates were analyzed by immunoblotting with indicated antibodies.

Supplementary Fig. S6

a

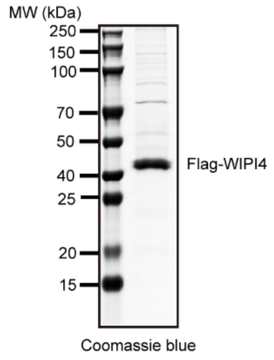

Supplementary Fig. S6: The purification of Flag-WIPI4.

(a) Flag-WIPI4 purified from *E. coli* was analyzed by SDS-PAGE with Coomassie blue staining.

Supplementary Fig. S7

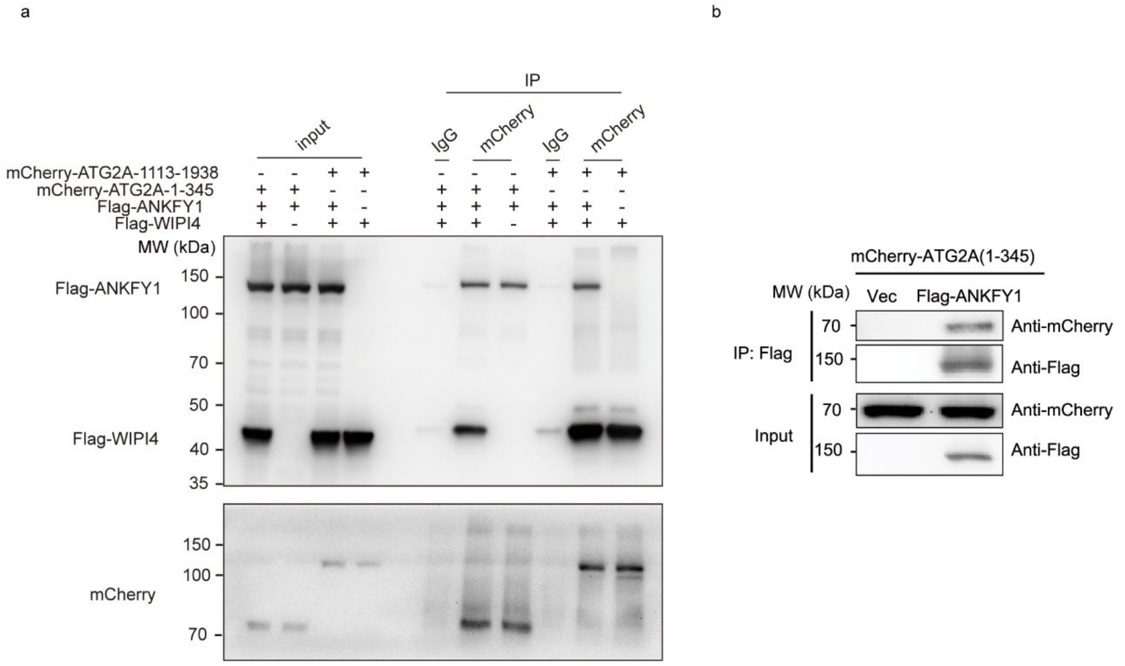

Supplementary Fig. S7: The N-tip of ATG2A binds with ANKFY1, while the C-tip binds with WIPI4.

(a) HEK293T cells transfected with indicated plasmids for 36 h, and then cells were subjected to IP and analyzed by Western blot. **(b)** HEK293T cells transfected with Flag-ANKFY1 and mCherry-ATG2A (1-345) as indicated for 36 h, and then cells were subjected to Flag IP and analyzed by Western blot.
